# Molecular Detection of *Encephalitozoon cuniculi* in Migratory Waterfowl of the Genus *Anser* (Anseriformes: Anatidae) in Poland

**DOI:** 10.3390/pathogens14050489

**Published:** 2025-05-16

**Authors:** Piotr Solarczyk, Agnieszka Perec-Matysiak, Agnieszka Wojtkowiak-Giera, Mike Heddergott

**Affiliations:** 1 Department of Biology and Medical Parasitology, Poznan University of Medical Sciences, 61-701 Poznan, Poland; psolar@ump.edu.pl (P.S.); awojtko@ump.edu.pl (A.W.-G.); 2 Department of Parasitology, University of Wrocław, 51-148 Wrocław, Poland; agnieszka.perec-matysiak@uwr.edu.pl; 3 Department of Zoology, Musée National d‘Histoire Naturelle, 2160 Luxembourg, Luxembourg

**Keywords:** encephalitozoonosis, microsporidia, genotyping, molecular epidemiology, zoonosis one health

## Abstract

Microsporidiosis is a zoonotic disease that derives from disparate sources. Most of the microsporidial agents are host-specific but some are capable of interspecies transmission, causing disease in various animals including humans. Human microsporidiosis may be caused by 17 species, with *Encephalitozoon cuniculi*, *E. intestinalis* and *E. hellem* mostly being responsible for human infections worldwide. Wildlife and migratory waterfowl can serve as reservoirs of these human-infectious agents and play a significant role in disseminating these pathogens into the environment. The aim of the study was to detect *E. cuniculi*, *E. intestinalis* and *E. hellem* in wild, migratory greater white-fronted geese (*Anser albifrons*) and other Anatidae members in feacal samples obtained in north-western Poland, using a molecular method. We collected 189 fecal droppings from Anatidae species (75 samples from greater white-fronted geese and 114 from other *Anser* spp.) during autumn migration. New species specific primers for PCR amplification were used to amplify a fragment of the small subunit ribosomal (SSU) rRNA of *E. cuniculi*, *E. intestinalis* and *E. hellem*. All fecal droppings were negative for *E. intestinalis* and *E. hellem* whereas *E cuniculi* was detected in 6 of 189 fecal samples (3.2%; 95% CI: 1.3–6.3%). In total, 1 of 75 tested fecal samples of greater white-fronted geese was positive (1.3%; 95% CI: 0.08–5.7%) while 5 of 114 (4.4%; 95% CI: 1.6–9.1%) tested fecal samples without exact species affiliation (only *Anser* sp.) were also positive. The phylogenetic analysis placed the sequences obtained from the birds’ droppings in the clade *E. cuniculi* from various rodents, wild carnivores and humans. Our results provide the first description of the occurrence and genotyping of the microsporidian *E. cuniculi* in greater white-fronted geese and in other members of the Anserinae Subfamily. Our findings support the results of other authors that *E. cuniculi* may originate from diverse sources, including common waterfowl. Our results are important in a One Health context, as wild migrating waterfowl may disseminate this zoonotic agent in remote regions through their migratory behaviour. These species should be considered significant sources of zoonotic pathogens, potentially hazardous to domestic and farmed animals as well as humans.

## 1. Introduction

Microsporidians (Microsporidia) are diverse, fungus-related obligate and intracellular pathogens. They have been reported from a wide range of host species belonging to protozoa and cold- and warm-blooded metazoa, including humans [1]. The spores of their infective stages have the ability to survive outside the host cell and have been found world wide in various habitats, including freshwater, brackish water, and marine and terrestrial environments [2]. Most of the 1700 described species are reported to infect only a single host, but those that are generalists can infect a large number of hosts and play a role as zoonotic sources of microsporidiosis [3]. In humans, the infection can be caused by 17 species from 220 genera including *Encephalitozoon*, *Anncaliia*, *Pleistophora*, *Trachipleistophora*, *Vittaforma*, *Endoreticulatus*, *Microsporidium* and *Nosema*. These microsporidians can cause a variety of clinical manifestations ranging from gastrointestinal disorders, such as enteritis, to diffuse systemic infections without specific manifestations in immunocompetent and immunocompromised individuals [4].

Currently, opportunistic *Encephalitozoon cuniculi*, *E. intestinalis* and *E. hellem* infections from disparate sources are mainly responsible for human microsporidiosis [5]. Since *Encephalitozoon* spillover is possible into unrelated and distant hosts, the role of such host is relevant in a One Health context [6,7]. Wild animals are relevant zoonotic pathogens disseminators, that transmit intestinal agents mainly via contaminated water sources to humans [8,9]. Based on recent data, the presence of the zoonotic *E. bieneusi*, *E. hellem* and *E. cuniculi* has been confirmed in many species of domestic, captive and wild populations of waterbirds worldwide [10,11,12]. One of the most important reservoirs for microsporidia is large migratory birds (e.g., the family Anatidae), which can spread infectious spores to remote regions, with Central Europe being a natural stopover habitat [8,13].

Reports of human microsporidiosis in Poland are rare. There are reports presenting microsporidia infections in patients with immunodeficiency. According to polymerase chain reaction (PCR) results, *E. bieneusi* and *E. cuniculi* were confirmed in these hospitalized individuals [14]. In other research, two species as *E. cuniculi* and *E. intestinalis* were confirmed in three patients using the fluorescence in situ hybridization (FISH) method in Poland [15].

Studies on microsporidiosis in wild animals in Poland are rare. So far, evidence of *E. bieneusi* has been reported in red foxes (*Vulpes vulpes*) and the two invasive species raccoon dog (*Nyctereutes procyonoides*) and raccoon (*Procyon lotor*) [16,17]. Another study in southwestern Poland confirmed the infection of various wild mouse species with *E. bieneusi* [18]. Epidemiological surveys on microsporidia also prove infections in various mammals from zoos. Captive animals from zoological gardens were positive for *E. bieneusi* [19]. Microsporidian DNA has also been confirmed in invertebrate hosts and one of the dominant kinds found in Culicidae hosts was *E. hellem* [1].

Birds have so far only been the subject of one study on microsporidia in Poland, where *E. intestinalis* and *E. hellem* were detected in wild and captive birds [8]. Evidence of *E. cuniculi* in wild animals, including geese, has so far been lacking in Poland.

Alongside environmental studies, modern surveillance of human-infectious microsporidia includes the examination of various wild and domestic animal species, including migratory waterfowl [20]. In terms of human microsporidia presence in wild birds associated with human-shared environments, wild waterfowl may become an important source of endemic microsporidiosis foci establishment. During migration, birds arrive in the open areas of national parks and stay there for extended periods of time. During this time, they are usually undisturbed by humans (e.g., by visitors, birdwatchers or farmers), which may lead to significant surface pollution where their feces accumulate [13]. The aim of the study was therefore to use molecular methods to detect microsporidia in wild, migratory greater white-fronted geese (*Anser albifrons*) and other members of the genus *Anser* in fecal samples obtained in north-western Poland

## 2. Material and Methods

### 2.1. Study Area

**Figure 1:**
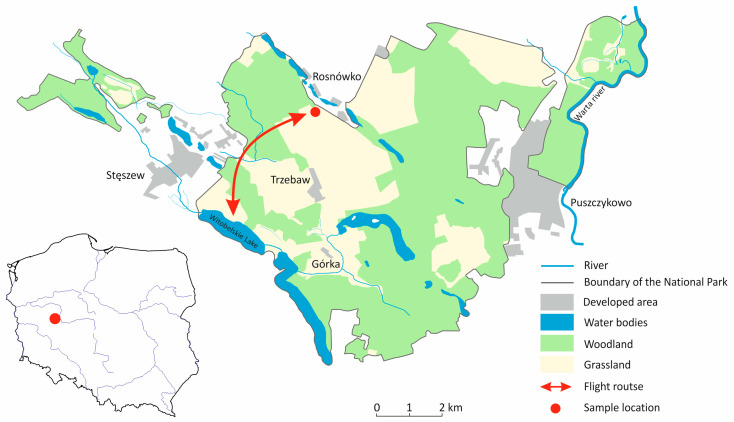
Map of the Wielkopolska National Park (WNP) and its location in Poland. The map shows the activities of geese between Lake Witobelskie for resting and the agricultural areas for foraging within the WNP.

The Wielkopolski National Park (Polish: Wielkopolski Park Narodowy; WPN) is a lowland nature reserve in western Poland, located approximately 15 km southwest of the city of Poznan (Figure 1). The WPN is surrounded by several large (Luboń, Mosina, Puszczykowo, Stęszew) and many small villages. There are also several small settlements within the park itself. The WPN has a large number of lakes of various sizes as well as some periodic reservoirs [21]. The WPN was founded in 1957 and expanded in 1996 and currently covers an area of 7619.8 ha, whith a buffer zone of 7383.2 ha. The entire area of the WPN is part of the Ostoja Wielkopolska Natura 2000 nature reserve and serves as one of Poland’s most important resting places for migratory geese (>10,000). It is thus a bird sanctuary of international importance [22].

### 2.2. Sample Collection

During the 2020–2021 autumn migrations, 189 fresh fecal samples were collected in the WPN, in a field near Rosnówko (16°45′34″ E/52°17′27″ N) in the Greater Poland Voivodeship, Poland (Figure 1). The field was visited four times and fecal samples were collected. The greater white-fronted geese arrive in October and form a monospecific flock until early November. During this period, we visited the field twice to collect 75 (40%) fecal samples of this species. The classification of the samples was easy as only fresh droppings were collected and only this goose species used the field for foraging. The peak of the number of different Anserine species was recorded in December 2020 and January 2021. Besides greater white-fronted geese, the collected flock consisted of greylag geese (*Anser anser*) and tundra bean geese (*Anser serrirostris*) (Table 1). During this period, we collected a further 114 (60%) fecal samples. Flock size was estimated visually using binoculars. To avoid possible contamination, only fresh fecal samples were collected immediately after the geese had flown away. Furthermore, care was taken to ensure that no foreign bodies such as soil or plant parts contaminated the droppings samples. Bird activity covered the areas, including Lake Witobelskie and its surroundings as well as remote parts of farmland and farmland within the WNP, to which birds had unrestricted access (Figure 1).

### 2.3. Molecular Analysis

About 5–10 g of fecal material was collected from the ground, placed into plastic containers and stored at 4 °C in a portable fridge. Total DNA was extracted using the QIAamp DNA Stool Mini Kit (Qiagen, Hilden, Germany) according to the manufacturer’s instructions, except for an overnight incubation with Proteinase K, and elution in 50 μL of elution buffer. DNA was stored at −20 °C.

We used Oligo software v. 7.60 (DBA Oligo, CA, USA) to design new *Encephalitozoon* specific PCR primers for the ampification of a small fragment of the small ribosomal subunit (SSU) rRNA of *E. cuniculi*, *E. intestinalis* and *E. hellem* (see Supplementary Materials Figure S1). The first set of primers MicunF (5′-ATAGTGGTCTGCCCCTGTG-3′) and MicunR (5′-GTCTTCGCATTTCACCTCTCG-3′) (amplicon size 439 bp) were designed by using a published GenBank sequences of *E. cuniculi* (Acc. no. L17072). The next set of primers MiinF (5′-GACGGCTCAGTGATAGTACG-3′) (amplicon size 420 bp) and MiinR (5′-AATCCCCCAAACAAAGACATACA-3′) (amplicon size 458 bp), was designed on the basis of a published GenBank sequences of *E. intestinalis* (Acc. no. U09929), whereas the pair of MihelF (5′-AGGTAAGTTCTGGGGGTGGT-3′) and MihelR (5′-CAGTCAGGGTCTTCGTATTTC-3′) (458 bp) was established on the basis of a published GenBank sequences of *E. hellem* (Acc. no. L19070). Polymerase chain reactions were performed in a total volume of 20 μL and included AmpliTaq Gold Fast PCR Master Mix UP, 0.6 μM of each primer and 2 μL of template DNA. Negative controls (master mix without template + water) were used in each reaction. DNA extracted from cultured microsporidian spores of the three commercial lines of microsporidian spores: *E. cuniculi* (P103C, 1 × 10/6), *E. hellem* (P103H, 1 × 10/6) and *E. intestinalis* (P103I, 1 × 10/6) (Waterborne Inc., New Orleans, LA, USA) were used as positive controls.

The specificity of the primers was verified by performing PCR using genomic DNA of the three commercial *Encephalitozoon* species (P103C *E. cuniculi*, P103H *E. hellem* and P103I *E. intestinalis*), *G. duodenalis* isolates belonging to assemblage A (Portland-1 isolate), assemblage B (HP-124 isolate) and *Cryptosporidium parvum* (cow isolate).

The PCR cycling conditions were as follows: 10 min at 94 °C, 45 cycles of 95 °C for 30 s, 56 °C (MicunF/R) or 55 °C (MihelF/R and MiinF/R) for 40 s, and 72 °C for 10 min. Amplicons were electrophoresed on 1% agarose gel and stained with Midori Green (EURx). Positive products were purified using the QIAquickPCR purification Kit (Qiagen, Hilden, Germany) according to the manufacturer’s instructions. The amplicons were sequenced using the ABI Prism 3130 XL BigDye v3.1, Terminator Cycle (Applied Biosystems, CA, USA) in both directions with the same set of primers. Trace files were checked and edited using BioEdit version 7.0.5.3. (http://www.mbio.ncsu.edu/BioEdit/bioedit.html (10 May 2024)) software. Contigs were edited and manually assembled in GeneDoc v. 2.7.000 [23] and compared with deposited sequences in GenBank. A phylogenetic tree was constructed by the Maximum Likelihood algorithm and distance-based analyses were conducted using alignments obtained from ClustalW using MEGA version 11 [24]. Bootstrap proportions were calculated by analysis of the 1000 replicates of the phylogenetic tree.

### 2.4. Statistical Analysis

All principal statistical analyses were conducted following the guidelines outlined by Zar [25]. Prevalence, the percentage interpreted as the probability of finding positive samples in gathered probes and confidence intervals (95% CI) were calculated using R software R v.4.0.2 [26]. The graphics were prepared and provided with information from various publications and finalized with CorelDRAW 2021 (Corel, Ottawa, ON, Canada).

## 3. Results

**Figure 2:**
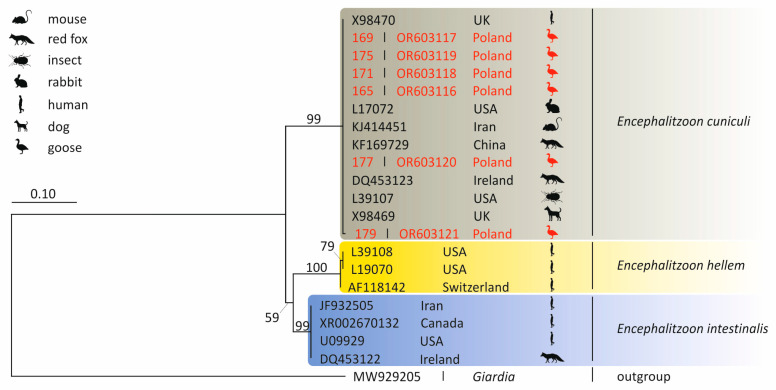
Phylogenetic tree of the SSU rRNA gene region nucleotide sequences of *Encephalitozoon cuniculi* isolates obtained in Poland. The tree was constructed by using the Kimura2-parameter model and is in the units of the number of base substitutions per site. The *Giardia* sequence represents an outgroup.

*Encephalitozoon cuniculi* DNA was detected in six fecal samples (n = 189; 3.2%; 95% CI: 1.3–6.3%). One greater white-fronted goose samples was positive (n = 75; 1.3%; 95% CI: 0.08–5.7%), while five samples from the multispecies flock were also positive (n = 114; 4.4%; 95% CI: 1.6–9.1%) (Table 2). All faecal droppings were negative for *E. intestinalis* and *E. hellem*. The alignment of the five *E. cuniculi* sequences (isolates 165, 169, 171, 175, and 177) showed 100% identity with a sequence from a rabbit isolate (Acc. no. L17072). The sequence of *E. cuniculi* isolate 179 differed at two SNPs (Single Nucleotide Polymorphisms) compared to the same rabbit *E. cuniculi* isolate. Phylogenetic analysis of the SSU rRNA nucleotide sequences obtained from the microsporidia-positive isolates placed them in a clade alongside sequences obtained from rodents, wild carnivores and humans (Figure 2).

Correct amplicons were obtained only when primer pairs designed to detect the species-specific DNA of *Encephalitozoon* species were used. The MicunF/MicunR, MihelF/MihelR and MiinF/MiinR primers were successfully used to amplify the DNA belonging to *E. cuniculi* (P103C), *E. hellem* (P103H), and *E. intestinalis* (P103I) isolates, respectively. The designed primers proved to be specific because they did not cross-amplify with the DNA of *G. duodenalis* belonging to assemblages A and B and *C. parvum*.

The sequences from this study have been submitted to GenBank under unique accession numbers: isolate 165 (Acc. no. OR603116), isolate 169 (Acc. no. OR603117), isolate 171 (Acc. no. OR603118), isolate 175 (Acc. no. OR603119), isolate 177 (Acc. no. OR603120) and isolate 179 (Acc. no. OR603121).

## 4. Discussion

To date, in addition to the main host of *E. cuniculi* (rabbit, *Oryctolagus cuniculus*), the presence of these microsporidia has been successfully demonstrated in many other reservoirs of rodents, birds, domestic, farm and wild animals, including humans, worldwide [27,28,29,30,31,32,33,34,35]. As the transmission of *E. cuniculi* to unrelated and distant hosts is possible, the role of such hosts is discussed in the context of One Health [6,7]. Wildlife, including waterfowl, are important spreaders of zoonotic agents that transmit enteric pathogens to humans mainly via contaminated water sources [8,9].

Currently, molecular approaches are the most efficient methods for detecting microsporidia in various samples. Like many other studies, we relied on molecular assays to detect and identify *Encephalitozoon* species [33,36,37,38]. Using species-specific primers for conventional PCR, this study demonstrated that greater white-fronted geese and other anserine species in Poland are infected with *E. cuniculi*. By targeting a fragment of the small subunit ribosomal (SSU) rDNA, we detected *E. cuniculi* DNA in a sample deposited by greater white-fronted geese on the ground during migration. This sampling approach allowed us to confidently attribute the detected DNA to greater white-fronted geese, as these birds formed flocks exclusively with members of their species before other Anserinae species appeared in the field. We also detected the genetic material of *E. cuniculi* in five other fecal samples, but could not associate the pathogen with a specific host species as the monitored waterfowl colony consisted of greylag geese, tundra bean geese and greater white-fronted geese.

The detection of *E. cuniculi* in any member of the Anserinae would be a first. Previously, *E. cuniculi* DNA and spores were detected in wild white storks (*Ciconia ciconia*), great cormorants (*Phalacrocorax carbo*) and great crested grebe (*Podicepes cristatus*) in Slovakia [39]. In Poland, only one species of domestic waterfowl, *Anser anser domestica*, has been reported as positive for *E. intestinalis* [40]. To our knowledge, the greater white-fronted goose is a newly identified host for *E. cuniculi*. The overall infection rate of *E. cuniculi* in the three waterfowl species in Slovakia was higher (40.4%) compared to our results (3.2%), which might be due to the higher sensitivity of the real-time PCR used by Malčeková et al. [39]. In addition, the broad host range of *E. cuniculi* makes the pathogen the most common microsporidian species in Slovakia, where it was detected in both animals and humans [29,41]. However, our results contrast with those of a study in Poland on free-ranging and domestic geese, in which *E. hellem* was primarily detected in waterfowl such as mallards (*Anas platyrhynchos*), greylag geese and mute swans (*Cygnus olor*) [8,40].

*Encephalitozoon cuniculi* has been classified into four genotypes (I–IV), identifiable by 5′-GTTT-3′ repeats within the internal transcribed spacer (ITS) molecular marker. Despite this genetic diversity, *E. cuniculi* exhibits low host specificity, underscoring its zoonotic potential [2,42,43,44]. Our phylogenetic analysis suggests that migrating geese may harbor and disseminate zoonotic microsporidia through their feces.

*Encephalitozoon cuniculi* spores can survive for up to six weeks or longer after being excreted into the environment, particularly under humid conditions and suitable temperatures [45]. The natural transmission route of *E. cuniculi* between individuals involves the ingestion of infective stages present in the environment, shed in feces—even from asymptomatic hosts [38,46,47,48]. Among *Encephalitozoon* species, *E. cuniculi* is notably prevalent in wild rodents, particularly within the murine and arvicoline subfamilies. In Japan, infection rates have reached 32% in Japanese field mice (*Apodemus speciosus*), 18.9% in Japanese grass mice [*Microtus montebelli* (=*Alexandromys montebelli*)], and 12.5% in field mice (*Apodemus argenteus*) [11]. Similar infection rates have been found in wild mice across three European countries, with the highest rates observed in yellow-necked mice (*Apodemus flavicollis*) at 21% and striped field mice (*Apodemus agrarius*) at 23.6%—in environments shared by humans, wild rodents, and waterfowl, including cities, reserves, and recreational areas [12,17,38,47].

Microsporidia excreted in feces can potentially be transmitted to new hosts. The likelihood of infection may be higher in areas where individuals use or share water resources. Lakes are commonly used by geese as resting places. They feed on land during the day and then return in flocks to rest on the water, often away from the shore, at night. One possible transmission route for microsporidia is water. Some studies have reported the presence of *E. bieneusi* and *E. intestinalis* in surface water and groundwater, respectively [49,50]. Although no reports of *E. cuniculi* in water bodies exist worldwide, our results suggest that waterfowl may contribute to water contamination, particularly during migration when they sometimes form large flocks. All goose species examined in our study utilized a shared water source, Lake Witobelskie, as a resting place.

**Figure 3:**
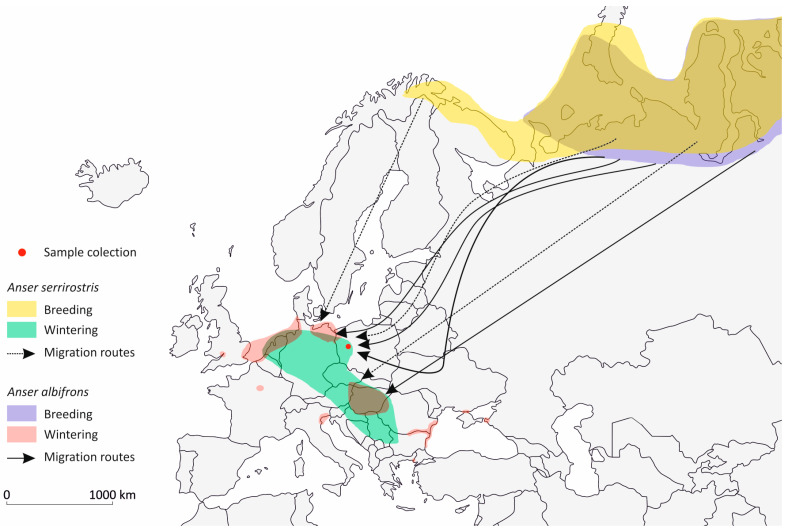
Breeding and wintering areas of tundra bean goose (*Anser serrirostris*) and greater white-fronted goose (*Anaser albifrons*) including their main migration routes with in Europe (simplified after Kruckenberg et al. [54], Fox et al. [57]).

As geese migrate in large flocks, their presence during the migration period can have a regional impact on the local environment. They leave large amounts of feces in the water and soil habitats they use [51], increasing the likelihood of fecal contamination in water bodies and fields during their migration [39,40,52]. Studies on the migration patterns of the *Anser* species we investigated show that the greylag goose is now primarily a short-distance migrant, and in some regions, a resident bird within Europe [53]. For the greylag goose, breeding and wintering areas often overlap under favorable climatic conditions. In contrast, the greater white-fronted goose and tundra bean goose are long-distance migrants, with their breeding areas in the Arctic clearly separated from their wintering grounds in Central Europe [54,55,56,57]. Based on findings from ringing and satellite telemetry studies, we have created a migration map for the greater white-fronted goose and the tundra bean goose (Figure 3) to illustrate how *E. cuniculi* could spread [54,57]. These migration routes of these two species once again illustrate the large catchment area during the migration period in fall and spring and the associated distribution of infected feces.

Interestingly, the agricultural land management in the study area within the WNP has remained unchanged in recent years, providing a favorable environment for migrating geese to utilize foraging habitats, which in turn has a significant impact on local ecosystems [58]. The presence of *E. cuniculi* DNA in goose fecal droppings found in arable fields used for crop production suggests a potential risk of infection for farm animals that are traditionally fed raw agricultural products. All goose species included in the study use remote areas for feeding during migration, so, aside from professional workers, there is a low probability of human infection through contact with bird feces in the national park. However, shared spaces, such as city parks or other suburban environments frequented by migrant birds, may pose a more obvious link to human health risks [9]. Although direct *E. cuniculi* transmission to humans from wild geese has yet to be proven, the main exposure of this micropathogen is via an indirect route through contaminated water during migration time. Lakes and ponds in sub-urban areas or urban public parks are the most direct links to the risk to human health.

## 5. Conclusions

Microsporidia are still an enigmatic and understudied group of eukaryotic pathogens with a global distribution. They appear to be more prevalent in free-ranging animals than previously thought. Although *E. cuniculi* infections can cause a range of systemic symptoms, particularly in immunocompromised individuals, this *Encephalitozoon* species should be considered a public health concern due to its significant adaptability to various animal hosts, which can spread it across broad geographical areas [59,60,61]. We designed new sets of primers to detect three *Encephalitozoon* species using PCR. The sets of primer pairs used in this study did not match the SSUr DNAsequence of isolates belonging to gastrointestinal protozoa from *Giardia* and *Cryptosporidium*. In conclusion, the use of *Encephalitozoon* species-specific primers with further sequencing of the amplification products designed in this study allows the quick and reliable identification of human-infectious microsporidia in goose droppings. This action allows us to detect three microsporidian species that differ in their epidemiology. Our findings support those of other researchers, confirming that *E. cuniculi*, with its broad host specificity, may originate from diverse sources, including common waterfowl. Given the zoonotic risk for individuals who may come into direct contact with birds (e.g., ornithologists, animal keepers, or farmers), personal hygiene practices should be emphasized. Our study fills a gap in the data by evaluating *E. cuniculi* in wild birds, which may disseminate this zoonotic agent to remote regions through their migratory behavior. In our opinion, targeted research is needed to determine the risk of environmental contamination by wild geese with *E. cuniculi* during migration.

## Figures and Tables

**Table 1:** The number of fecal samples collected from observable flocks of Anatidae during the autumn migration period in Wielkopolska National Park (Rosnówko), Poland.

Year	Month	No. of Samples Instead	No. of Samples Instead	Flock Size (Number of Spotted Birds)
2020	October (31.10.2020)	35		39 *A.albifrons*
	November (06.11.2020)	40		47 *A. albifrons*
	December (25.12.2020)		20	298 *A. anser* 97 *A. serrirostris* 91 *A. albifrons* 9 *Anser* spp.
2021	January (17.01.2021)		94	510 *A. serrirostris* 370 *A. albifrons* 198 *A. anser* 93 *Anser* spp.
Total		189		

**Table 2:** The number of positive samples obtained during autumn migration from monospecies flocks consisting only of geater white-fronted geese (*Anser albifrons*) and multi-species flocks, which included greylag geese (*Anser anser*) and tundra bean geese (*Anser serrirostris*) alongside greater white-fronted geese in Wielkopolska National Park (Rosnówko), Poland.

Year	Month	No. of Samples/Positive in Monospecies Flock	No. of Samples/Positive in Multi-Species Flock	Isolate No.
2020	October	35/0		
	November	40/1		165
	December		20	
2021	January		94/5	169, 171, 175, 177, 179
Negative/positive		75/1	114/5	
Total negative/positive		189/6		

## Data Availability

The original contributions presented in the study are included in the article. Further inquiries can be directed to the corresponding author.

## Supplementary Materials

The following supporting information can be downloaded at: https://www.mdpi.com/article/10.3390/pathogens14050489/s1, Figure S1: Complete sequences of small subunit ribosomal RNA gene derived from *E. hellem*, *E. intestinalis* and *E. cuniculi* deposited in GenBank.
