# Supplementary material for: Molecular Detection of Encephalitozoon cuniculi in Migratory Waterfowl of the Genus Anser (Anseriformes: Anatidae) in Poland
Source: Pathogens. 2025 May 16;14(5):489. doi: 10.3390/pathogens14050489 (PMC12114933; doi:10.3390/pathogens14050489)
Supplement: Supplementary file 1 [file pathogens-14-00489-s001.zip › pathogens-3339658-supplementary.pdf]

Figure S1 Complete sequences of small subunit ribosomal RNA gene derived from *E. hellem*, *E. intestinalis* and *E. cuniculi* deposited in GenBank.

E. hellem, 1314 bp (Acc. no. L 19070)

CACCAGGTTGATTCTGCCTGACGTGGATGCTATTCTCTGGGGCTAAGCCATGCATGTTTATGAAGCCTTTATGGGGGATTG
ACGGACGGCTCAGTGATAGTACGATGATTTGATTGGGAGCCTGGATGTAACTGTGGGAAACTGC**AGGTAAGTTCTGGGG**
GTGGTAGTTTGTAGCTACTGCGTACCGAGTAAGTTGTAGGCCTATCAGCTGGTAGTTAGGGTAATGGCCTAACTAGGCGG
AGACGGGAGACGGGGGATCAGGGTTTGATTCCGGAGAGGGAGCCTGAGAGATGGCTACTACGTCCAAGGATGGCAGCA
GGCGCGAAACTTGCCTAATCCTTATTGGGGAGGCGGTTATGAGAAGTAAGATGTTTAGCAAGTATAAATTTGTTGTGATTT
ACTGGAGGGCAAGTCGGGTGCCAGCAGCCGCGGTAATACCTGCTCCAGTAGTGTCTATGGTGAATGCTGCAGTTAAAATG
TCCGTAGTTGTTTGTATGTCTTTTGAGTGATGTTTATGGTTTTTAGTGGATGTAGTTTTATTGTAGCAGAGGACGAGGGGCA
CTGGATAGTTGGGCGAGGGGT**GAAATACGAAGACCCTGACTG**GACGAAGAGAAGCGAAGGCTGTGTTCTTGGACTTTTG
TGGTGATGAAGGACGAAGGCTAGAGGATCGAAATCGATTAGATACCGTTTTAGTTCTAGCAGTAAACGATGCCGACTGGA
CGGGACTGTTTTAGTGTTGTCCGAGAGAAATCTTAAGTATGTGGGTTCTGGGGATAGTATGCTCGCAAGAGTGAAACTTG
AAGAGATTGACGGAAGGACACCACAAGGAGTGGAGTGTGCGGCTTAATTTGACTCAACGCGGGGCAACTTACCGGTTCT
GAAGTGAGTGTGAGAGTGTTTTTACATGATGCTTACGGCGGTGGTGCATGGCCGTTTTAAATGGATGGCGTGAGCTTTGG
ATTAAGTTACGTAAGATGTGAGACCCTTTTTGACTGTGCTCTATGGGGCAAGGGAGGAATGGAACAGAACAGGTCCGTTA
TGCCCTGAGATGAAGCGGGCGGCACGCGCACTACGATAGATGCCTATGTGGGCTACTGTGAGGGATGAAGCTGTGTAAT
GGGCTTCTGAACGTGGAATTCCTAGTAAGAATGATTGAACAAGTTATTTTGAATGTGTCCCTGTCCTTTGTACACACCGCCC
GTCGCTATCTAAGATGACGCAGTGGACGAAGATTGAGAGGTCTGAGTCTTTCGTGTTAGATAAGATATAAGTCGTAACAT
GGCTGCTGTTGGAGAACCATTAGCAGGATCATAA

E. intestinalis, 1295 bp (Acc. no. U 09929)

CACCAGGTTGATTCTGCCTGACGTGGATGCTATTCTCTGGGACTAAGCCATGCATGTTGATGAACCTTGTGGGGGATTGAC
G**GACGGCTCAGTGATAGTACG**ATGATTTGGTTGGCGGGAGAGCTGTAACTGCGGGAAACTGCAGGTAGGGGGCTAGGA
GTGTTTTTGACACGAGCCAAGTAAGTTGTAGGCCTATCAGCTGGTAGTTAGGGTAATGGCCTAACTAGGCGGAGACGGGA
GACGGGGGATCGGGGTTTGATTCCGGAGAGGGAGCCTGAGAGATGGCTACTACGTCCAAGGATGGCAGCAGGCGCGAA
ACTTGCCTAATCCTTTGGGGAGGCGGTTATGAGAAGTGAGTTTTTTTCGAGTGTAAAGGAGTCGAGATTGATTGGAGGGC
AAGTCGGGTGCCAGCAGCCGCGGTAATACCTGCTCCAATAGTGTCTATGGTGAATGCTGCAGTTAAAAAGTCCGTAGTCTT
T**TGTATGTCTTTGTTTGGGGGATT**ATGTCCTGATGTGGATGTAAGAGGTTTGGCAGAGGACGAGGGGCACCGGATAGTT
GGGCGAGGGGTGAAATACGAAGACCCTGACTGGACGGACAGAAGCGAAGGCTGTGCTCTTGGACTTATGTGACGATGAA
GGACGAAGGCTAGAGGATCGAAATCGATTAGATACCGTTTTAGTTCTAGCAGTAAACGATGCCGACTGGACGGGACTATA
TAGTGTTGTGCATGAGAAATCTTGAGTATGTGGGTTCTGGGGATAGTATGCTCGCAAGAGTGAAACTTGAAGAGATTGAC
GGAAGGACACCACAAGGAGTGGAGTGTGCGGCTTAATTTGACTCAACGCGGGGCAACTTACCGGTTCTGAAGCGGGCAG
GAGAACGAGGACGGGATGCGCGCGGCGGTGGTGCATGGCCGTTTGAAATGGATGGCGTGAGCTTTGGATTAAGTTGCGT
AAGATGTGAGACCCTTTGACAGTGCTCTTTGGGGCAAGGGAGGAATGGAACAGAACAGGTCCGTTATGCCCTGAGATGA
AGCGGGCGGCACGCGCACTACGATAGATGGCGAGGGAGCCTGCTGTGAGGGATGAAGCTGTGTAATGGGCTTCTGAAC
GTGGAATTCCTAGTAATAACGATTGAACAAGTTGTTTTGAATGGGTCCCTGTCCTTTGTACACACCGCCCGTCGCTATCTAA
GATGACGCAGTGGACGAAGATTGGAAGGTCTGAGTCCTTCGTGTTAGATAAGATATAAGTCGTAACATGGCTGCTGTTGG
AGAACCATTAGCAGGATCATAA

E. cuniculi, 1299 bp (Acc. no. L 17072)

CACCAGGTTGATTCTGCCTGACGTGGATGCTATTCTCTGGGGCTAAGCCATGCATGCTTGTGAACTCTTTGTGGGGGATTA
GCGGACGGCTCAGTGATAGCACGATGATTTGTTTGCGGGATGAGCAGTAGCTGCGGGAAACTGCA**GATAGTGGTCTGCC**
CCTGTGGGGGTTGGCAAGTAAGTTGTGGGCCTATCAGCTGGTAGTTAGGGTAATGGCCTAACTAGGCGCAGACGGGATA
CGGGGGATCAGGGTTTGGTTCCGGAGAGGGAGCCTGAGAGATGGCTACTACGTCCAAGGATGGCAGCAGGCGCGAAAC
TTGCCTAATCCTTTGGGGAGGCGGTTATGAGAAGTGATGTGTGTGCGAGTGCAAAGGGGTCGCATGTGATTGGAGGGCA
AGTCGGGTGCCAGCAGCCGCGGTAATACCTGCTCCAATAGTGTCTATGGTGGATGCTGCAGTTAAAATGTCCGTAGTCTGT
TGTGTATGTCTTTGTGTGTGATGTTTGTGGTTGTGTGTGGATGTAGTGATGTGTGTGGCAGAGGACGAGGGGCACTGGAT

AGTTGGG**CGAGAGGTGAAATGCGAAGAC**CCTGACTGGACGAGCGGAAGCGAAGGCTGTGCTCTTGGACTAATGTTGCG
ATGAAGGACGAAGGCTAGAGGATCGAAATCGATTAGATACCGTTTTAGTTCTAGCAGTAAACGATGCCGACTGGACGGG
ACAGTGTGTGTTGTCCATGAGAAATCTTGAGTATGCGGGTTCTGGGGATAGTATGCTCGCAAGAGTGAAACTTGAAGAGA
TTGACGGAAGGACACCACAAGGAGTGGAGTGTGCGGCTTAATTTGACTCAACGCGGGGCAACTTACCGGCTCTGAAGGA
TGCCTGTGAGTGCATGGCATGAGGCATGCGGCGGTGGTGCATGGCCGTTTTAAATGGATGGCGTGAGCTTTGTCTTAAGT
TGCGTAAGATGTGAGACCCTTTGACGGTGTTCTACGGAGCAAGGAGGGGATGGAAGAGAACAGGTCCGTTATGCCCTGA
GATGAGGCGGGCTGCACGCGCACTACGATAGATGGCGCTTCTGCCTGCTGTGAGGGATGAAGCTGTGTAAGGGGCTTCT
GAACGTGGAATTCCTAGTAATAGCGGCTGACGAAGCTGCTTTGAATGTGTCCCTGTCCTTTGTACACACCGCCCGTCGCTA
TCTAAGATGACGCACTGGACGAAGATCGGAAGGTCTGAGTCCTGAGTGTTAGATAAGATATAAGTCGTAACATGGCTGCT
GTTGGAGAACCATTAGCAGGATCATA
